# Plant microRNAs from *Moringa oleifera* Regulate Immune Response and HIV Infection

**DOI:** 10.3389/fphar.2020.620038

**Published:** 2021-02-11

**Authors:** Antonella Minutolo, Marina Potestà, Valentina Roglia, Marco Cirilli, Federico Iacovelli, Carlotta Cerva, Joseph Fokam, Alessandro Desideri, Massimo Andreoni, Sandro Grelli, Vittorio Colizzi, Rosario Muleo, Carla Montesano

**Affiliations:** ^1^ Department of Biology, University of Rome “Tor Vergata”, Rome, Italy; ^2^ Department of Agricultural and Forestry Science, University of Tuscia, Viterbo, Italy; ^3^ Department of Agricultural and Environmental Sciences, University of Milan, Milan, Italy; ^4^ Department of System Medicine, University of Rome “Tor Vergata”, Rome, Italy; ^5^ Chantal BIYA International Reference Centre for Research on HIV/AIDS Prevention and Management, Yaoundé, Cameroon; ^6^ Department of Experimental Medicine, University of Rome “Tor Vergata”, Rome, Italy; ^7^ Faculty of Sciences and Technology, Evangelic University of Cameroon, Bandjoun, Cameroon

**Keywords:** HIV, post-transcription regulation, *Moringa oleferia*, cross-kingdom, immune response

## Abstract

Traditional medicine is often chosen due to its affordability, its familiarity with patient’s cultural practices, and its wider access to the local community. Plants play an important role in providing indispensable nutrients, while specific small RNAs can regulate human gene expression in a cross-kingdom manner. The aim of the study was to evaluate the effects of plant-enriched purified extract microRNAs from *Moringa oleifera* seeds (MO) on the immune response and on HIV infection. Bioinformatic analysis shows that plant microRNAs (*p*-miRs) from MO belonging to 18 conserved families, including *p-*miR160h, *p*-miR166, *p-*miR482b, *p-*miR159c, *p-*miR395d, *p-*miR2118a, *p-*miR393a, *p-*miR167f-3p, and *p-*miR858b are predicted to target with high affinity BCL2, IL2RA, TNF, and VAV1, all these being involved in the cell cycle, apoptosis, immune response and also in the regulation of HIV pathogenesis. The effects of MO *p*-miRs transfected into HIV+ PBMCs were analyzed and revealed a decrease in viability associated with an increase of apoptosis; an increase of T helper cells expressing Fas and a decrease of intracellular Bcl2 protein expression. Meanwhile no effects were detected in PBMCs from healthy donors. In CD4^+^ T cells, transfection significantly reduced cell activation and modified the T cell differentiation, thereby decreasing both central and effector memory cells while increasing terminal effector memory cells. Interestingly, the *p*-miRs transfection induces a reduction of intracellular HIV p24 protein and a reduction of viral DNA integration. Finally, we evaluated the effect of synthetic (mimic) *p*-miR858b whose sequence is present in the MO *p-*miR pool and predicted to target VAV1, a protein involved in HIV-Nef binding. This protein plays a pivotal role in T cell antigen receptor (TCR) signaling, so triggering the activation of various pathways. The transfection of HIV+ PBMCs with the synthetic *p-*miR858b showed a reduced expression of VAV1 and HIV p24 proteins. Overall, our evidence defines putative mechanisms underlying a supplementary benefit of traditional medicine, alongside current antiretroviral therapy, in managing HIV infection in resource-limited settings where MO remains widely available.

## Introduction

MiRNAs are a short sequence of non-coding small RNAs that play a pivotal role in gene regulation at the post transcriptional level, in mammals and in plants. It has been demonstrated that a cross-kingdom interaction can occur between the plant-derived miRNAs and their “host,” through diet (Liang et al., 2015; Teng et al., 2018). In 2012 it was reported for the first time that plant-derived miRNAs from rice passing through the gastrointestinal tract could be absorbed and reach, via the bloodstream, the body organs, thus exerting their biological function (Zhang et al., 2012). Even though it remains an issue still much debated (Campbell, 2020), there is increasing evidence on the regulatory role of gene expression machinery in host cell by intake and bioavailability of dietary plant-derived miRNAs (Liang et al., 2015; Zhou et al., 2015; Lukasik and Zielenkiewicz, 2017; Minutolo et al., 2018). *Moringa oleifera* Lam. (MO) is a medicinal plant, used for centuries in traditional medicine due to its richness in essential nutrients (Popoola and Obembe, 2013; Matic et al., 2018). Moreover MO-based preparations are scientifically documented as being anti-inflammatory, antihypertensive, antimicrobial, antioxidant, and antidiabetic (Ojewole, 2006; Anwar et al., 2007; Dhongade et al., 2017; Bhattacharya et al., 2018) along with other plants used in traditional medicine (Sagnia et al., 2014). Furthermore, MO improves hepatic and renal functions; it also regulates thyroid hormones, and stimulates the immune system, protecting against oxidative stress, inflammation, hepatic fibrosis, liver damage, hypercholesterolemia and cancer (Stohs and Hartman, 2015; Almatrafi et al., 2017). MO bioactivity depends mainly on the presence of different classes of plant secondary metabolites (Kou et al., 2018; Saucedo-Pompa et al., 2018). In 2016 MO miRNome has been sequenced, showing the presence of several conserved miRNAs (Pirrò et al., 2016b). Many of the MO miRNAs are conserved across multiple plant species; some of these miRNAs were predicted to interact with multiple target genes in the mammalian cells, with potential therapeutic implication for human diseases (Pirrò et al., 2016a; Minutolo et al., 2018; Potestà et al., 2019). More recently we reported that MO seeds aqueous extract (MOES) contain *p*-miRs, that were also characterized (Pirrò et al., 2016b). Moreover, we demonstrated that the MOES was able to modulate proliferation and apoptosis in cancer cells; this ability was associated with the presence *p*-miRs, that were able to modulate these processes at a post-transcriptional level (Minutolo et al., 2018; Potestà et al., 2019; Potestà et al., 2020). Considering the complex interactions between the immune system and HIV, the present paper aims to investigate the effects of MO seed extracts enriched in miRNAs, on the regulation of immune response and on HIV replication and integration on peripheral blood mononuclear cells (PBMCs) from HIV-positive patients.

## Materials and Methods

### Patients Enrollment and Cohort Characterization

Thirty-five chronic HIV-infected subjects naïve to the combination antiretroviral treatment (cART-naïve) were enrolled in an open study by the Division of Clinical Infectious Diseases, Department of System Medicine, University of Rome “Tor Vergata.” Ethical approval for the collection and use of human samples was obtained in 2014 from the ethics committee of “Tor Vergata” Hospital, protocol number 15/14 (D.M.08.02.2013 D.G.R.146/2013; D.D.G.467 del 25.07.2013). The 35 HIV+ subjects were characterized for their immunological and virological status (Table 1 and Supplementary Figure S1) and their PBMCs used for the *ex vivo* experiments. The PBMCs from 30 healthy donors (HDs) were obtained from thirty individuals attending the local blood transfusion unit of Policlinico “Tor Vergata” in Rome. All HDs provided written informed consent. The PBMCs were separated by centrifugation gradient according to standard methods (Ficoll).

**TABLE 1:** HIV + individuals enrolled (n = 35).

Sex (F/M)	4/31		
	Mean	± SD	Reference values
Age (years)	38.6	11.86	
Viral load (CP/ml)	221,811	398,432.03	
CD3^+^CD4^+^ (%)	23.19	11.50	31–60
CD3^+^CD4^+^ (mmc)	354.16	231.92	410–1,590
CD3^+^CD8^+^ (%)	54.79	14.42	13–48
CD3^+^CD8^+^ (mmc)	828.74	528.38	190–1,140
CD4^+^/CD8^+^ ratio	0.5767	0.46	1.5–2.5
CD16^+^/CD56^+^ (%)	12.34	9.74	5–27
CD16^+^/CD56^+^ (mmc)	143.25	76.64	90–590
CD19^+^ (%)	8.62	4.98	6–25
CD19^+^ (mmc)	127.7	96.38	90–660

### Bioinformatics Analysis

The bioinformatic analysis was performed on a list of miRNAs (Supplementary Table S1) found in the MOES (Potestà et al., 2019), derived from MO miRNome, belonging to the most conserved plant families (Pirrò et al., 2016b). A support vector machine (SVM) classifier trained using an experimentally validated set of miRNA-mRNA interactions was used as a prediction tool. The detailed description of the *sklearn. svm.SVC* classifier implementation is reported in the Supplementary Material (Supplementary Data). For pathways and network analysis, the Metascape online tool (http://metascape.org) was used to identify the predominant biological processes and networks regulated by MO *p*-miRs. Briefly, following the identification of each gene, the expression values from the total gene expression data profile were extracted from the repository. The gene list was divided into two groups: a list of selected genes involved in immune response and inflammation, and a list of genes down-regulated by the *p*-miRs. Subsequently, an enrichment analysis was performed to identify the significant biological processes and networks modulated by *p*-miRs. The DAVID online tool (https://david.ncifcrf.gov/) was used to perform functional annotation clustering, using reference database of human complex diseases and disorders (Table 2) Genetic Association Database (GAD_DISEASE) at *p*-value cut of point (*p* < 0.05).

**TABLE 2:** Disease enriched analysis associated to *p-*miRs modulated genes (DAVID tool).

	Disease	n. genes	*p* Value	Bonferroni	Benjamini
1	HIV	29	2.14*E* − 16	4.13*E* − 13	1.38*E* − 13
2	Asthma	25	6.06*E* − 09	1.17*E* − 04	2.92*E* − 05
3	Cell lymphoblastic leukemia-lymphoma	22	1.73*E* − 06	3.33*E* − 03	6.67*E* − 04
4	Meningeal neoplasms/meningioma	22	2.35*E* − 06	4.53*E* − 02	7.54*E* − 04
5	Hyperreactivity/hypersensitivity, immediate	17	1.99*E* − 04	3.84*E* − 02	5.49*E* − 03
6	Lung cancer	28	4.30*E* − 04	8.29*E* − 02	1.04*E* − 01
7	Multiple sclerosis	28	9.09*E* − 05	1.75*E* + 01	1.95*E* − 01
8	Chronic obstructive pulmonary disease	24	1.30*E* − 01	2.51*E* + 02	2.28*E* − 03
9	Chorioamnionitis/fetal membranes, premature rupture	16	5.63*E* − 02	2.14*E* + 02	1.79*E* + 01
10	Premature/pre-eclampsia/premature birth	18	1.21*E* + 00	2.14*E* + 02	1.64*E* + 02
11	Lymphoma, large B-cell, diffuse	13	1.24*E* − 01	2.14*E* + 02	1.53E + 01
12	Tuberculosis	15	1.67*E* + 00	4.28*E* + 02	2.85*E* + 01
13	Hematologic/premature birth/skin diseases	16	1.97*E* + 00	4.28*E* + 02	2.68*E* + 02
14	Inflammation/premature birth	16	1.97*E* + 00	4.28*E* + 02	2.68*E* + 02
15	Rupture/infection of amniotic sac and membranes	17	2.68*E* + 01	5.14*E* + 03	3.02*E* + 03
16	Bladder cancer	24	2.91*E* − 01	5.57*E* + 02	3.09*E* + 02
17	Diabetes, type 1	17	3.71*E* + 00	7.07*E* + 03	3.72*E* + 03
18	Hypersensitivity	11	3.82*E* + 00	7.28*E* + 03	3.64*E* + 03
19	Infection/inflammation/premature birth	15	4.26*E* − 01	8.14*E* + 03	3.88*E* + 03

### MO *p-*miRs Pool Extraction, Characterization, and Transfection

MO seeds were provided by the Cameroonian Association of Traditional Practitioners. MO *p*-sR pool was extracted from the aqueous extract of MO seeds by NucleoSpin® miRNA kit (MACHEREY-NAGEL, Germany) as previously described (Potestà et al., 2019). The presence of the most conserved *p-*miRs (Supplementary Table S1) was evaluated by RT-qPCR, as previously reported (Pirrò et al., 2016b; Minutolo et al., 2018); relative quantity of *p-*miRs was quantified by the 2^−ΔΔCt^ method, where 5S rRNA was used as a housekeeping gene. To evaluate the effects of *p-*miRs, PBMCs from HIV+ subjects and from HDs were stimulated by IL2 (20 U/ml, Sigma-Aldrich, St. louis, MO) for 72 h in RPMI 1640 (Life Technologies, Grand Island, NY) supplemented with 10% FCS (Life Technologies), 2 mM glutamine, 50 IU/ml penicillin, and 50 IU/ml streptomycin (Hyclone, Cramlington, United Kingdom). PBMCs were transfected with the pool of *p*-miRs at concentrations of 1 μg/ml, this performed according to the lipofectamine (HF) method (Hi-Fect, Qiagen, HF) as previously described (Minutolo et al., 2018). As control for the treatment, the PBMCs were treated with the transfection vehicle (HF) alone. After 72 h, cells were harvested, washed twice in PBS, and assessments of viability, apoptosis and immunological analysis were performed.

### Transfection and Detection of Mimic *p-*miR858b

The PBMCs from 15 HIV+ participants and 15 HDs were transfected via the lipofectamine method (Hi-Fect, Qiagen German, HF) using 5 nM synthetic mimic *p*-miR858b (Invitrogen, United States) following the manufacturer’s instructions. For treatment, the mimic *p*-miR858b, methylated at the 3′ end (feature of plant-derived miRNAs) was used. The *p-*miR858b is present in several plant species included *Moringa oleifera*, *Malus domestica*, and *Arabidopsis thaliana* (Supplementary Figure 2). The mimic *p-*miR858b FITC-conjugated was used to evaluate the transfection efficacy. The *p*-miR858b FITC-conjugated positive cells, were observed by fluorescence microscopy, after 72 h from transfection (Evos Floid Cells Imaging Station, ThermoFisher Scientific, United States) and by flow cytometry (CytoFLEX, Beckman Coulter, United States). The presence and quantity of mimic *p*-miR858b, in PBMCs from HIV+ and HDs, were detected on Bio-Rad thermal cycler (IQ5) according to the instructions of EXIQON pre-designed primers (*p-*miR858b 5′ UUC​GUU​GUC​UGU​UCG​ACC​UGA-3′). For RNA, the isolation was performed using NucleoSpin RNA II (Machenery-Nagel, Dueren, Germany) according to the manufacturer’s instructions. Relative quantity was calculated by the 2^−ΔΔCt^ method, using 5S rRNA as housekeeping gene.

### Cell Viability and Apoptosis Assays

Cell viability and mortality rates were assessed by a 10% Trypan blue (EuroClone S.p.A., Italy) exclusion test after 72 h of treatment. Apoptosis was assessed by flow cytometry analysis, using a CytoFLEX (Beckman Coulter, United States), on isolated nuclei stained with Propidium Iodide (PI) (Merck KGaA, Germany). Data acquisition and analyses were performed using CytExpert 2.0 (Beckman Coulter, United States) on a minimum of 150,000 events for each sample.

### Immunostaining and Flow Cytometry Analysis of Cellular Proteins

For the analysis of T cells cluster of differentiation markers (CDs), approximately 1 × 10^6^ cells were suspended in 100 µl of PBS, incubated with anti-human CD95 FITC/CD197-CCR7 PE/CD8 pc5.5/CD45RA APC/CD4 APC Alexa700/CD3 APC Alexa750 antibodies (Beckman Coulter, United States) at 4°C for 30 min (Bordoni et al., 2019). In some experiments, the cells were incubated with anti-human FITC CD25-Fas or TNF-alpha (Beckman Coulter, United States). For Bcl2 intracellular protein expression the transfected PBMCs were harvested, fixed, permeabilized with 70% ethanol and incubated with PE-conjugated anti-human Bcl2 (BD Biosciences, United States). The intracellular protein expression of HIV-p24 was detected using a PE anti-HIV p24 antibody (Abcam, United Kingdom). All the stained cells were analyzed via CytoFLEX (Beckman Coulter) and the CytExpert 2.0 software (Beckman Coulter).

### Western Blot Analysis of VAV1

Aliquots of 1 × 10^6^ cells, subjected to various experimental conditions, were lyzed and processed for western blot analysis, as standard protocol. The primary antibodies used were rabbit monoclonal antibodies directed against VAV1 protein and goat monoclonal anti-human Beta-actin (Santa Cruz Biotechnology, CA United States). The secondary antibodies used were anti-goat and anti-rabbit IgG chain specific conjugated to peroxidase for Western Blot detection and anti-rabbit IgG-PE for flow cytometry analysis (Calbiochem, Merck Millipore, Darmstadt, Germany).

### Evaluation of HIV Integration

HIV integration was evaluated as previously described (Matteucci et al., 2015). In summary, DNA isolations were performed using NucleoSpin Tissue XS (Machenery-Nagel, Dueren, Germany) according to the manufacturer’s instructions. The DNA from HIV-infected PBMCs was diluted in Tris 1 mM at 1,000 cell-equivalents/µl; a qualitative PCR was performed by 100 cell-equivalents of genomic DNA in standard conditions: 1 PCR Buffer, 2.5 mM MgCl2, 0.2 mM deoxynucleoside triphosphates mix, 1.25 U of Taq (all from Promega) and 10 pmol b-globin-specific primers (b-glob 1 and b-glob2) or 10 pmol Alu-gag-specific primers (Alu 5-TCC​CAG​CTA​CTC​GGG​AGG​CTG​AGG-3; gag 5-CTG​TGA​AGC​TTG​CTC​GGG​TC-3). PCR products were used as a template for real-time PCR to detect integrated viral DNA by using a specific primer for long terminal repeat (LTR): forward primer (5′-ATA​CCA​CAC​ACA​AGG​CTA​CTT​CC-3′) and reverse primer (5′-GCA​GGC​TCA​CAG​GGT​GTA​AC-3′). Each sample was analyzed in triplicate; a negative control (no template reaction) was included in each experiment, to check contamination. Each experiment was completed with a melting curve analysis and all primer pairs showed a single peak in the melting curve analysis, confirming the specificity of amplification and the lack of non-specific products and primer dimers. Real-time PCR results, obtained from five different experiments, were represented as a ratio of treated infected samples vs. infected control samples.

### Statistical Analysis

All data are presented as the mean values ± standard deviation (SD) from PBMCs of HDs (n = 30) and HIV+ participants (n = 35). Data analyses were performed using the SPSS statistical software system (version 17.0 for Windows, United States). Comparisons between treated and untreated cells for the results on the Trypan blue assay, apoptosis assay, Bcl2, CD95, TNF-alpha and HIV-p24 intracellular protein expression were all conducted using t-test. For comparison of the means, the Bonferroni’s post-hoc multiple comparison ANOVA test was utilized. Significant differences are shown as *p < 0.05, **p < 0.01 and ***p < 0.001. For non-parametric correlations, a Pearson correlation coefficient was calculated.

## Results

### Computational Prediction of MO *p*-miRs Targeting Human Genes, and Network Analysis of the Processes Involved in Immune Response, Inflammation Pathways and HIV Replication/Integration by the Most Conserved MO *p*-miRs

**FIGURE 1:**
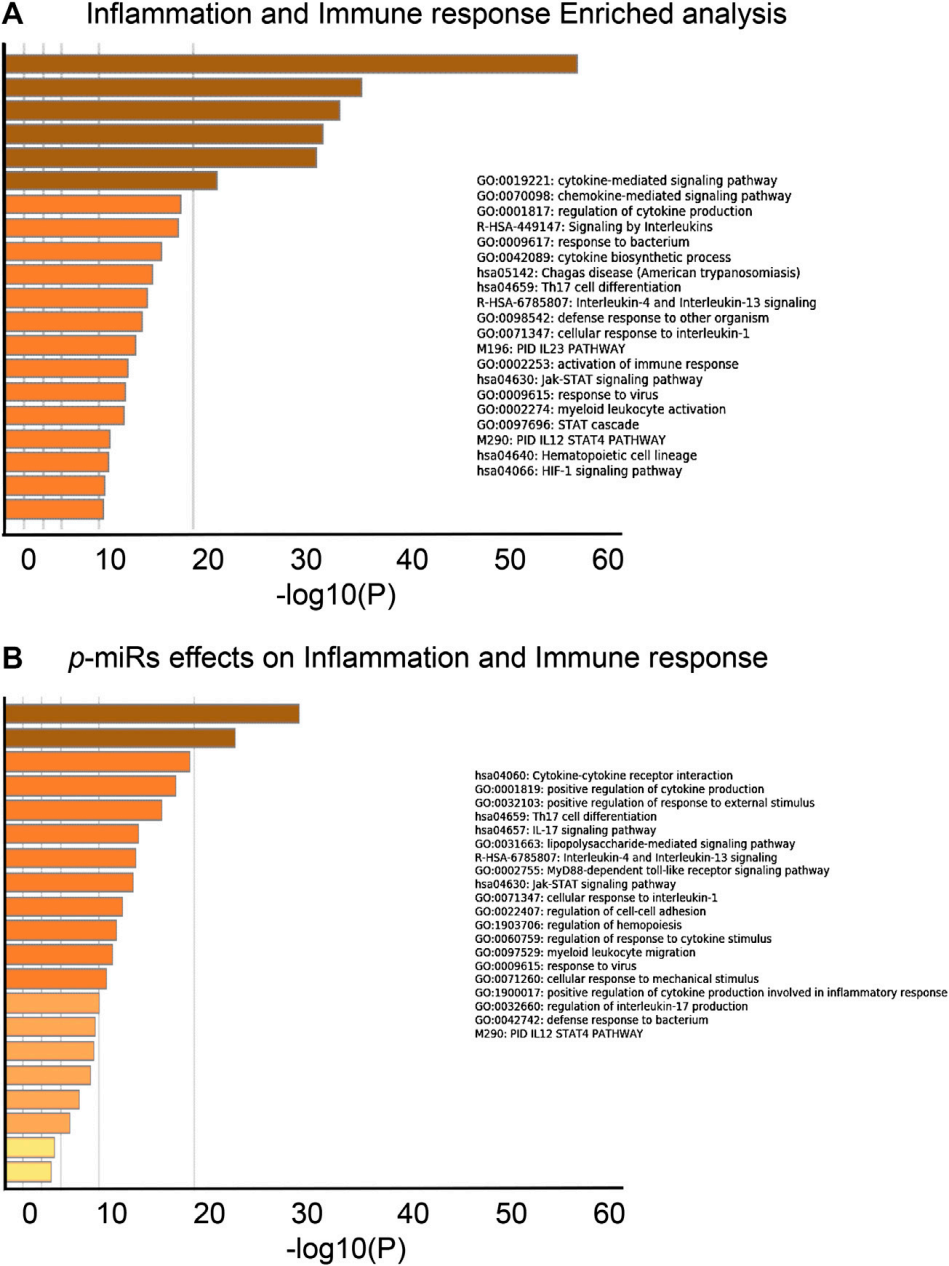
Biological processes enrichment analysis of putative *p*-miRs regulated genes. **(A)** Enrichment analysis related to the genes associated to immune response and inflammation. **(B)** Enrichment analysis related to the modulated gene by the most conserved MO *p*-miRs. Up to the top 20 enriched clusters are shown colored by *p*-values. All the statistically enriched terms were identified (GO/KEGG terms, canonical pathways, hall mark gene sets, etc.), accumulative hypergeometric *p*-values and enrichment factors were calculated and used for filtering. Then 0.3 kappa score was applied as the threshold to divide the tree into term clusters. Created by Metascape [http://metascape.org].

**FIGURE 2:**
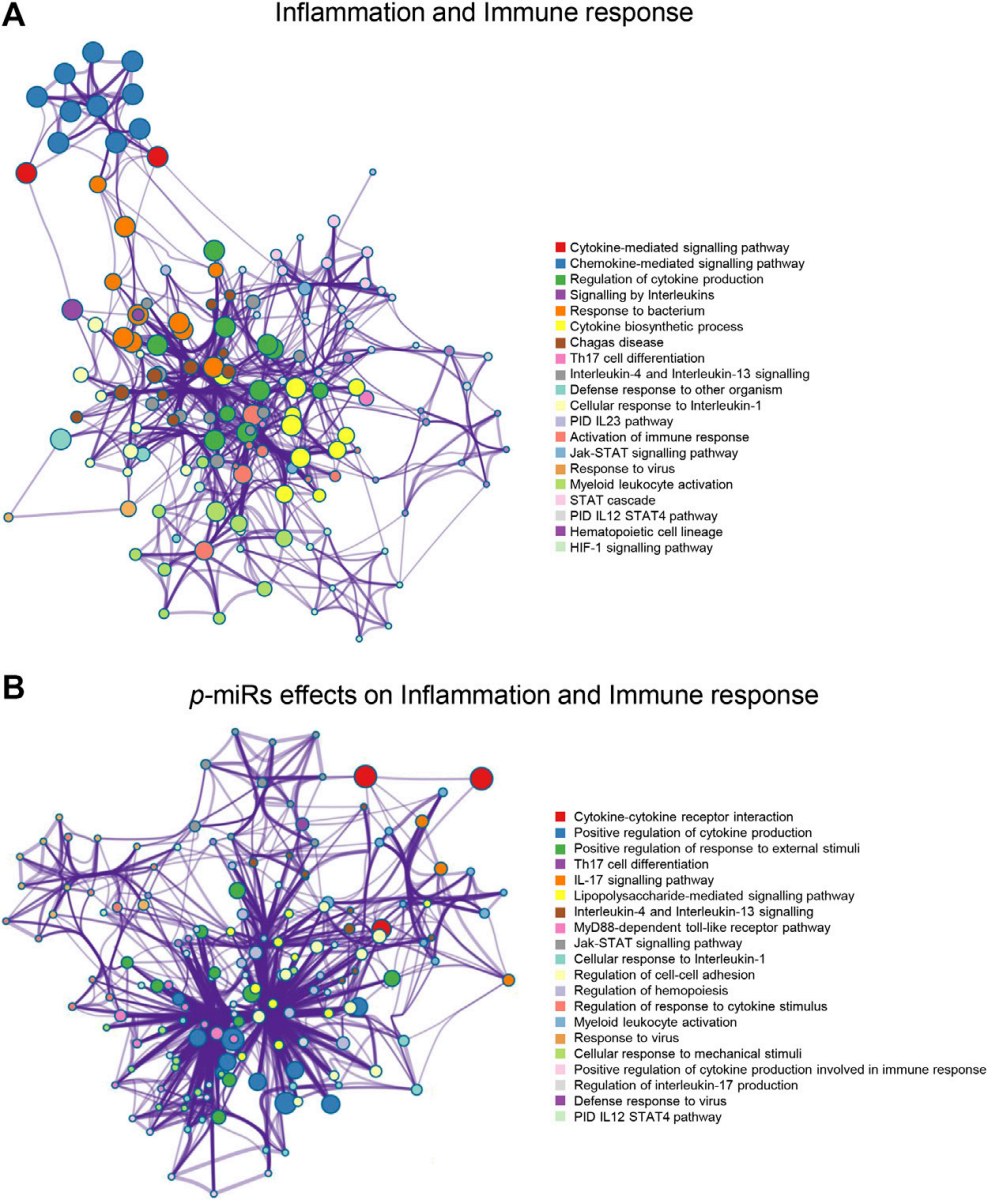
Enrichment network analysis for inflammation and immune response. **(A)** Networks layout of the clusters generated with the list of the genes associated with the inflammation and immune responses. **(B)** Networks layout of the clusters generated with the list of the genes regulated by *p*-miRs. Each circle node represents one enriched term, where its size is proportional to the number of input genes falling into that term, and its color represents its cluster identity (i.e. nodes of the same color belong to the same cluster). All similar terms with a Kappa similarity score >0.3 are connected by edges (the thicker the edge higher the similarity). One term from each cluster has been as a label. Created by Metascape [http://metascape.org].

A novel prediction tool obtained by combining different RNA-RNA interaction prediction algorithms was used in order to test the probability of the most conserved *p*-miRs to interact with the genes involved in HIV infection. All the conserved *p*-miRs were predicted to interact with a high probability for at least three genes (Supplementary Table S2). Interestingly, nine of these *p-*miRs (*p-*miR160h, *p-*miR166, *p-*miR482b, *p-*miR159c, *p-*miR395d, *p-*miR2118a, *p-*miR393a, *p-*miR167f-3p, and *p-*miR858b) were predicted to target BCL2, IL2RA, TNF, and VAV1, all with high affinity (Table 3). To better understand the significant biological processes, and the interconnections among the networks modulated by *p*-miRs, an analysis was generated by Metascape online tool (Zhou et al., 2019). The *p*-miRs were capable of binding 45 mRNAs associated with the immune response, 48 mRNAs of cytokines, chemokines, and their receptors. There were 94 possible targets of *p-*miRs associated with p53 and NfKb pathways. The enrichment analysis highlighted how *p*-miRs were potentially capable of significantly modifying important cellular processes associated with immune response and inflammation (Figure 1). Treatment with *p*-miRs showed modulation of genes related to biological processes: immune response, inflammation pathways, and response to infections (Figure 2).

**TABLE 3:** MO *p-*miRs targeting BCL2, IL2RA, TNF and VAV1.

	Plant-miRNA	Target Genes
1	miR160h	TNF (0.95)
		VAV1 (0.96)
2	miR166	BCL2 (0.93)
		IL2RA (0.87)
		VAV1 (0.96)
3	miR482b	BCL2 (0.97)
		VAV1 (0.93)
4	miR159c	IL2RA (0.98)
		TNF (0.98)
5	miR395d	BCL2 (0.91)
		VAV1 (0.96)
6	miR2118a	TNF (0.90)
7	miR393a	VAV1 (0.88)
8	miR167f-3p	TNF (0.94)
9	miR858b	VAV1 (0.95)

### Effects of *p*-miRs on Apoptosis in PBMCs from Naïve HIV+ Individuals and HDs

**FIGURE 3:**
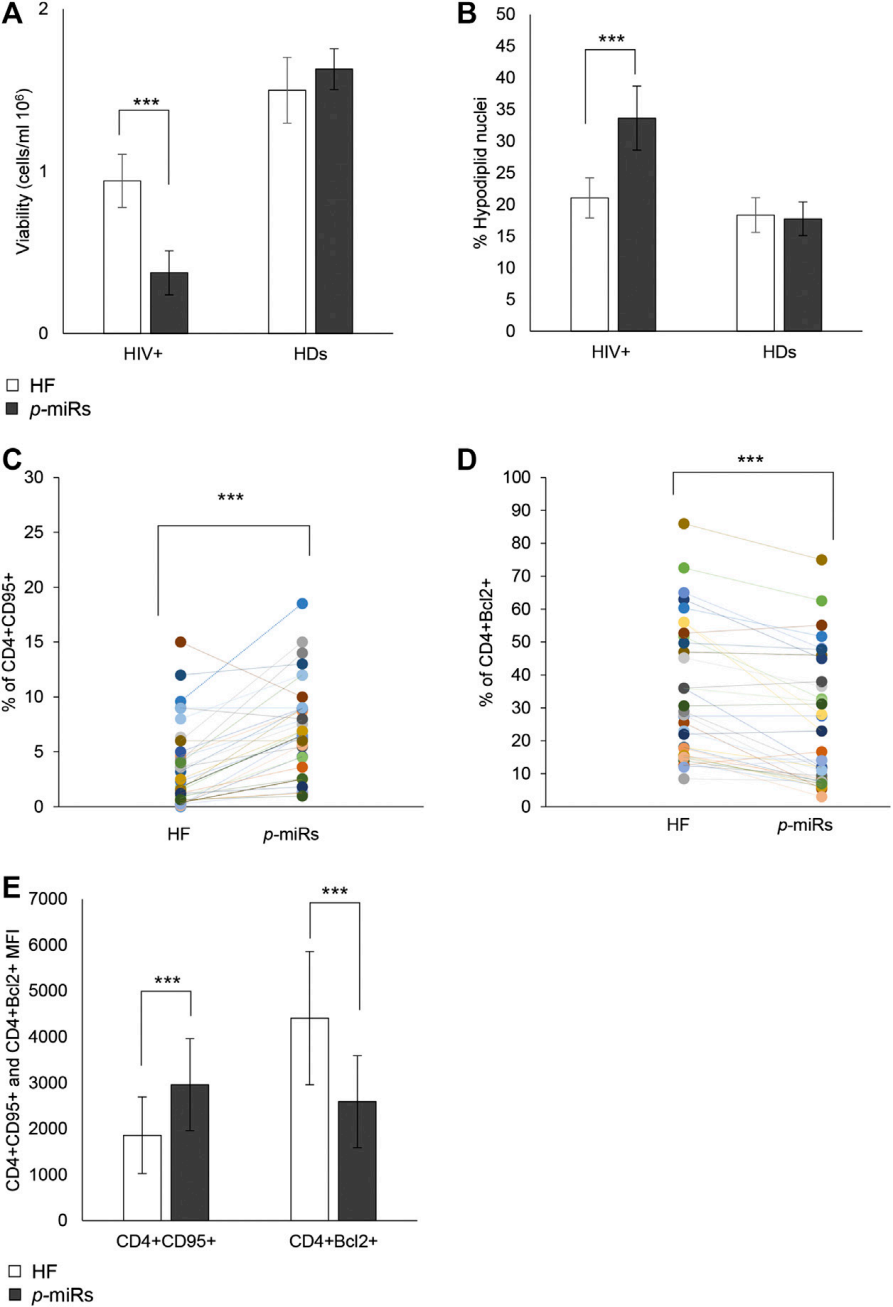
Effects of MO *p*-miR pool transfection in 35 HIV + patients and 30 HDs. **(A)** Number of viable cells analyzed by Trypan blue assay after *p-*miRs transfection in HIV + subjects and HDs. **(B)** Percentage of apoptotic cells after *p-*miRs transfection in HIV + subjects and HDs, evaluated by propidium iodide staining and flow cytometer analysis. **(C)** Percentage of CD4^+^ CD95^+^ cells in control and transfected T cells. **(D)** Percentage of CD4^+^ Bcl2^+^ cells in control and transfected T cells. **(E)** MFI of CD4^+^CD95^+^ T cells and of CD4^+^Bcl2^+^ T cells. Each colored dot, in the scatter plot **(D)** and **(C)** represents a different subject, control (HF) and treated (*p*-miRs). ***p < 0.001. All results derived from duplicate for each sample (n = 35 HIV+ and n = 30 HDs). Paired sample t-test was performed.

**FIGURE 4:**
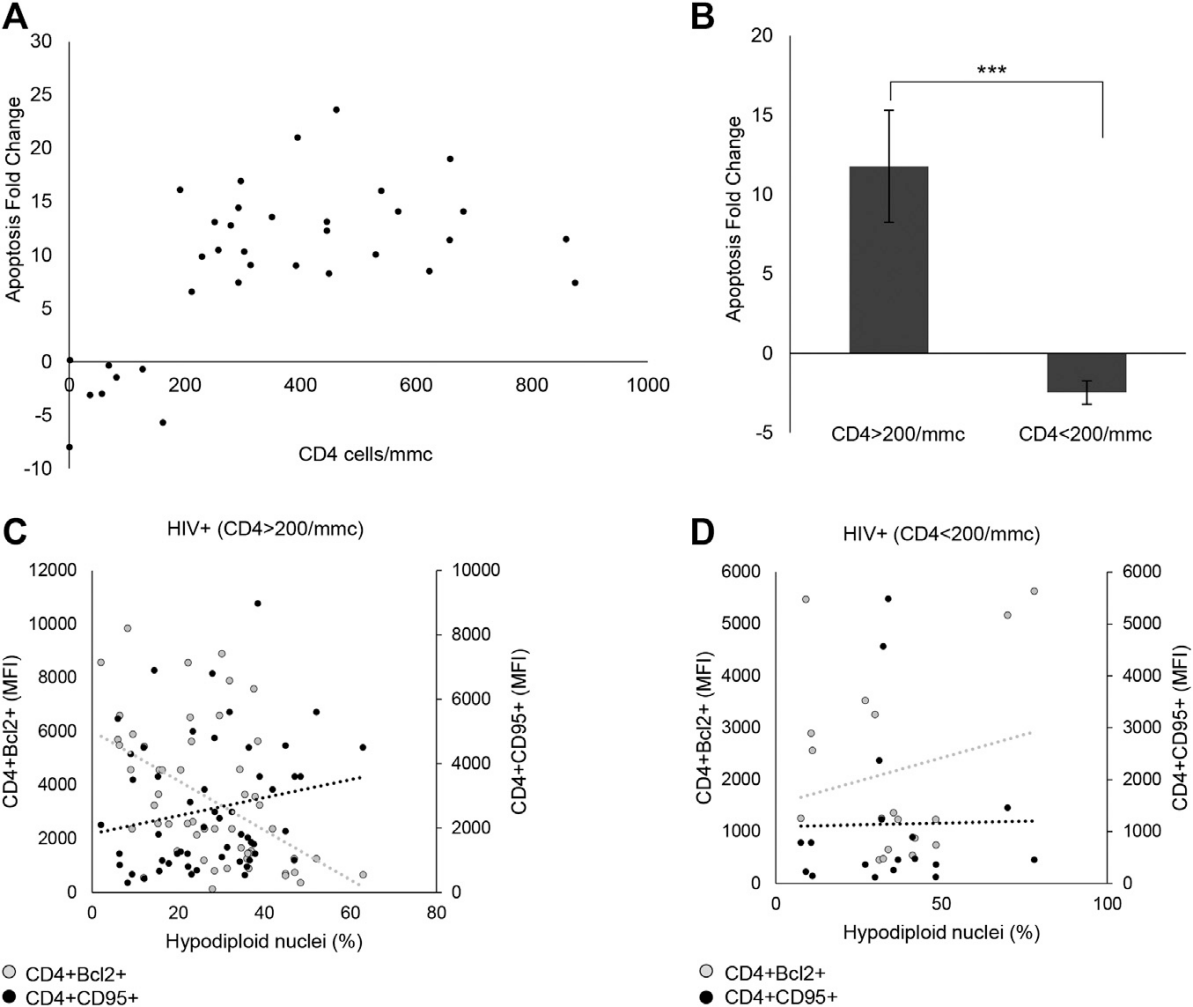
*p*-miRs effects on apoptosis and viability of CD4^+^ lymphocytes in HIV + subjects. **(A)** Correlation between apoptosis fold change and the absolute number of CD4^+^ lymphocytes, for each patient. **(B)** Apoptosis fold change in Responder (R) and Non-Responder (NR) subjects. **(C)** Correlation between Bcl2 MFI expression in CD4^+^ T cells (left Y axis) or CD95 MFI expression in CD4 T cells (right Y axis), and percentage of hypodiploid nuclei in R HIV + subjects, **(D)** and in NR HIV + subjects. ***p < 0.001. All results derived from duplicate for each sample (n = 35 HIV+). Nonparametric one-way ANOVA corrected with the Kruskal-Wallis test, and nonparametric Spearman’s *ρ* was used.

Through *ex vivo* experiments performed on the PBMCs from 35 HIV+ subjects and 30 HDs, the effect of *p*-miR pool transfection on the viability and apoptosis was evaluated, as well as the possible regulation of putative genes target involved in these pathways. 72 h after transfection, the *p*-miR pool determine a significant decrease in viability of PBMCs from HIV+ subjects analyzed by Trypan blue exclusion test, while the same treatment did not change the viability of PBMCs from HDs (Figure 3A). To understand whether the effect on viability was associated with the apoptosis induction, the cells were stained with PI 72 h after *p*-miRs transfection and were then analyzed by flow cytometry. As shown in Figure 3B, the *p*-miRs significantly enhanced the percentage of apoptosis in PBMCs from HIV+ subjects and did not affect the profile of PBMCs from HDs. The observed apoptosis was associated with both the increase of the percentage of CD4^+^ lymphocytes expressing CD95 and with the upregulation of its expression (Figures 3C,E). Conversely, the percentage of CD4^+^ lymphocytes expressing Bcl2 decreased following treatment, while the expression of Bcl2 was down-regulated (Figures 3D,E). As expected, PBMCs from HDs showed no susceptibility to *p*-miRs transfection. Nevertheless, among HIV+ cART-naïve participants, eight (8) out of 35 did not respond to MO *p*-miRs transfection (HIV+ Non-Responder: NR), while 27 responded with a significant increment in apoptosis after transfection (HIV+ Responder: R). Following stratification of participants as R *vs* NR, comparative analysis of absolute number of CD3^+^CD4^+^ cells revealed that only R with T cell CD4 counts equal or superior to 200/mmc responded to the MO *p*-miRs transfection; this is in contrast to the 8 NR who had a T cell CD4 count below 200/mmc (Figure 4A). A similar stratification based on CD3^+^CD4^+^ cell number (the difference in apoptotic cells, measured as fold change between R vs. NR) was statistically different (Figure 4B). This analysis was confirmed by a significant correlation between the percentage of hypodiploid nuclei and the expression of CD95 and Bcl2 proteins in R subjects. The correlation was positive in the case of increase in apoptosis and the number of CD4^+^ cells expressing CD95. Conversely, the correlation was negative in the case of number of CD4^+^ cells expressing Bcl2 (Figure 4C). These correlations analyses in NR subjects were not statistically significant (Figure 4D). Finally, the apoptosis of PBMCs from HDs, as well as from HIV+ individuals having CD4 cells lower than 200/mmc, was not influenced by the MO *p*-miR pool, showing that the treatment was able to induce a CD95-Fas and Bcl2 mediated apoptosis only in HIV+ subjects having higher CD4 T lymphocytes (>200/mmc).

### Effects of MO *p*-miRs on Activation and Differentiation Markers of CD4^+^ T Lymphocytes in HIV+ Individuals and HDs

**FIGURE 5:**
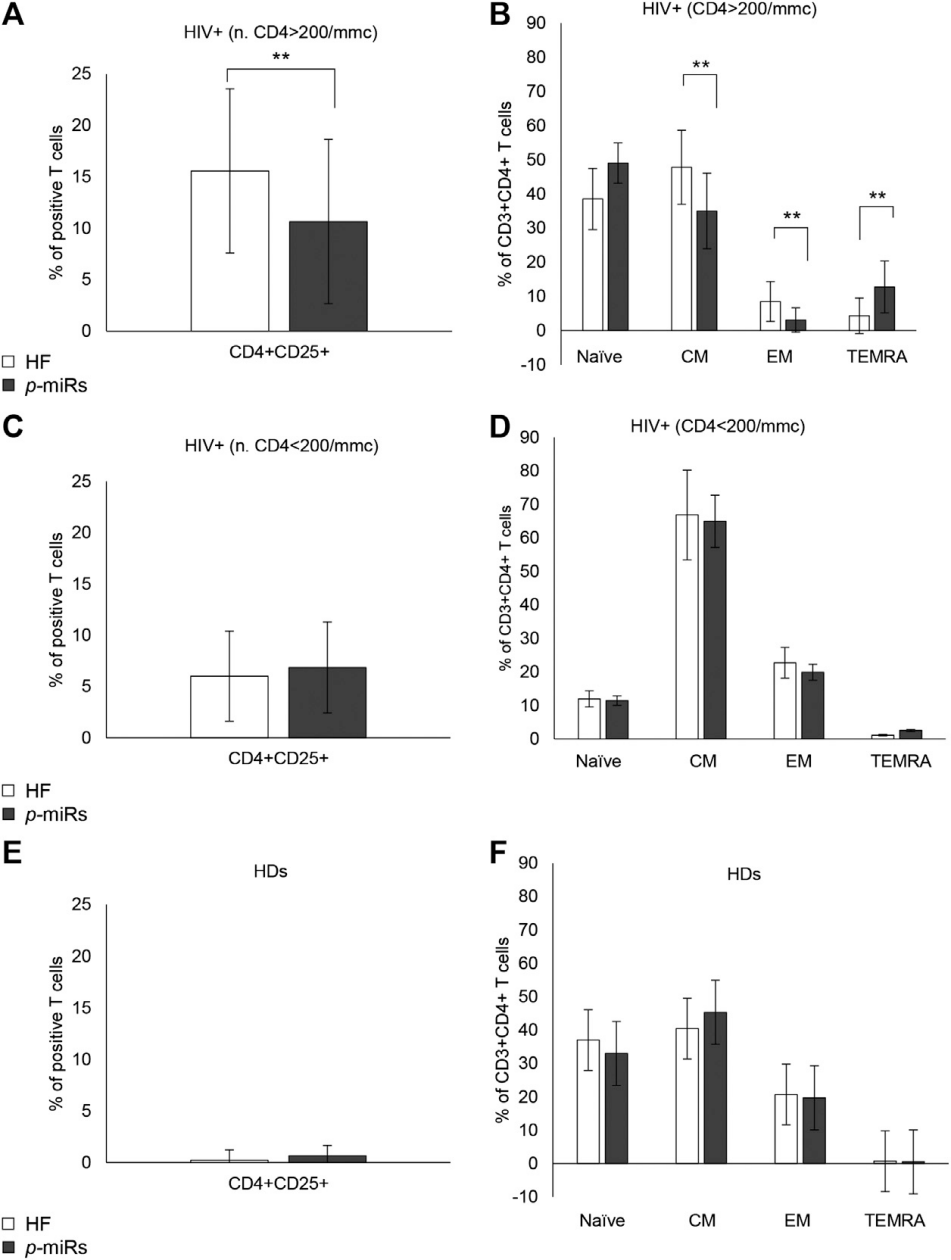
Cytofluorimetric analysis of activation and differentiation markers. **(A)** Percentage of positive CD4^+^CD25^+^ lymphocytes in R HIV+. **(B)** Percentage of positive CD3^+^CD4^+^ lymphocytes divided as Naïve (CCR7^+^CD45RA^+^), CM (CCR7^+^CD45RA^−^), EM (CCR7^−^CD45RA^−^), TEMRA (CCR7^−^CD45RA^−^), in R HIV + subjects. **(C)** Percentage of positive CD4^+^CD25^+^ lymphocytes in NR HIV + subjects. **(D)** Percentage of positive CD3^+^CD4^+^ lymphocytes, divided as Naïve, CM, EM, and TEMRA, in NR HIV + subjects. **(E)** Percentage of positive CD4^+^CD25^+^ lymphocytes in HDs **(F)** Percentage of positive CD3^+^CD4^+^ lymphocytes, divided as Naïve, CM, EM, and TEMRA, in HDs. **p < 0.01. All results derived from duplicate for each sample (n = 35 HIV+ and n = 30 HDs). Paired sample t-test was performed.

Among R HIV+ participants, the MO *p*-miRs transfection significantly reduced CD4 T cell activation and differentiation. Many phenotypic determinants have been associated with the course of HIV disease (Corneau et al., 2017), so highlighting the key role of T cell activation in driving the infection (Pardons et al., 2019). CD25 expression, evaluated by flow cytometry in CD3^+^CD4^+^ cells 72 h after transfection with the MO *p*-miRs, revealed that the treatment had a significant effect in reducing of CD4^+^CD25^+^ cells (Figure 5A), while no substantial effect was seen in NR subjects (Figure 5C). In HDs, no substantial change was observed (Figure 5E). Chronic untreated HIV infection has been associated with high T cell turnover and differentiation of T cells from the central memory (CM) to effector memory (EM) phenotype. The transfection of *p*-miRs modified the T cell differentiation patterns with a decline both in CM and EM cells (CCR7^+^CD45RA^−^ and CCR7^−^CD45RA^−^ respectively), and an increase in terminally differentiated effector memory (TEMRA, CCR7^−^CD45RA^+^) cells only in the population of R subjects (Figure 5B). In NR and in HDs no modification of differentiation markers was observed (Figures 5D,F).

### Effects of *p*-miRs on HIV Replication and Inflammation in PBMCs from R HIV+ Individuals

**FIGURE 6:**
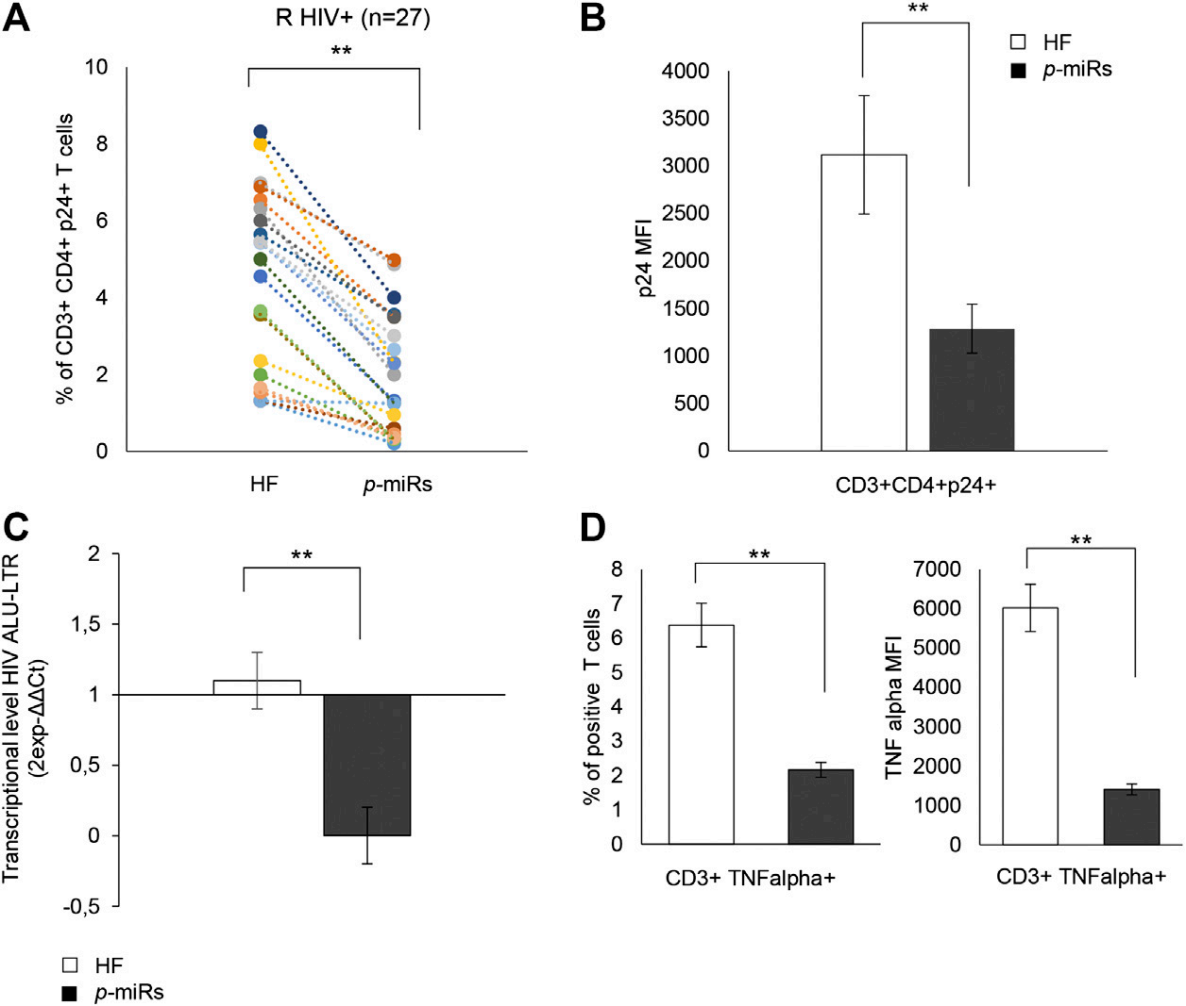
Effect of *p-*miRs on HIV p24 protein and TNF-alpha. **(A)** Percentage of CD3^+^CD4^+^ p24^+^ T cells after *p*-miRs transfection in all HIV + subjects analyzed. Each colored dot of the scatter plot represents a different subject, control (HF) and treated (*p*-miRs). **(B)** MFI of CD3^+^CD4^+^p24^+^ T cells after transfection with *p*-miRs. **(C)** Virus integration detected by PCR products used as template for real-time PCR to detect integrated viral DNA by using specific primer for LTR. **(D)** TNF-alpha percentage **(left)** and MFI **(right)** of positive T cells. **p < 0.01. All results derived from duplicate for each sample (n = 35 HIV+). Paired sample t-test was performed.

In R subjects, the ability of the MO *p-*miRs to interfere with viral replication was evaluated by monitoring intracellular p24. The treatment induced a significant decline in the percentage of CD3^+^CD4^+^ cells expressing HIV p24 (Figure 6A), and a reduction of the intracellular HIV p24 protein level. (Figure 6B). Additionally, PCR analysis showed that the treatment with MO *p-*miRs resulted in a significant decrease in virus integration (Figure 6C). The effect of MO *p*-miR pool on inflammatory processes revealed a significant reduction of the percentage of T cells expressing TNF-alpha, as well as a significant down regulation of this cytokine’s intracellular expression (Figure 6D).

### Effects of *p-*miR858b on VAV1 Expression and HIV Replication

**FIGURE 7:**
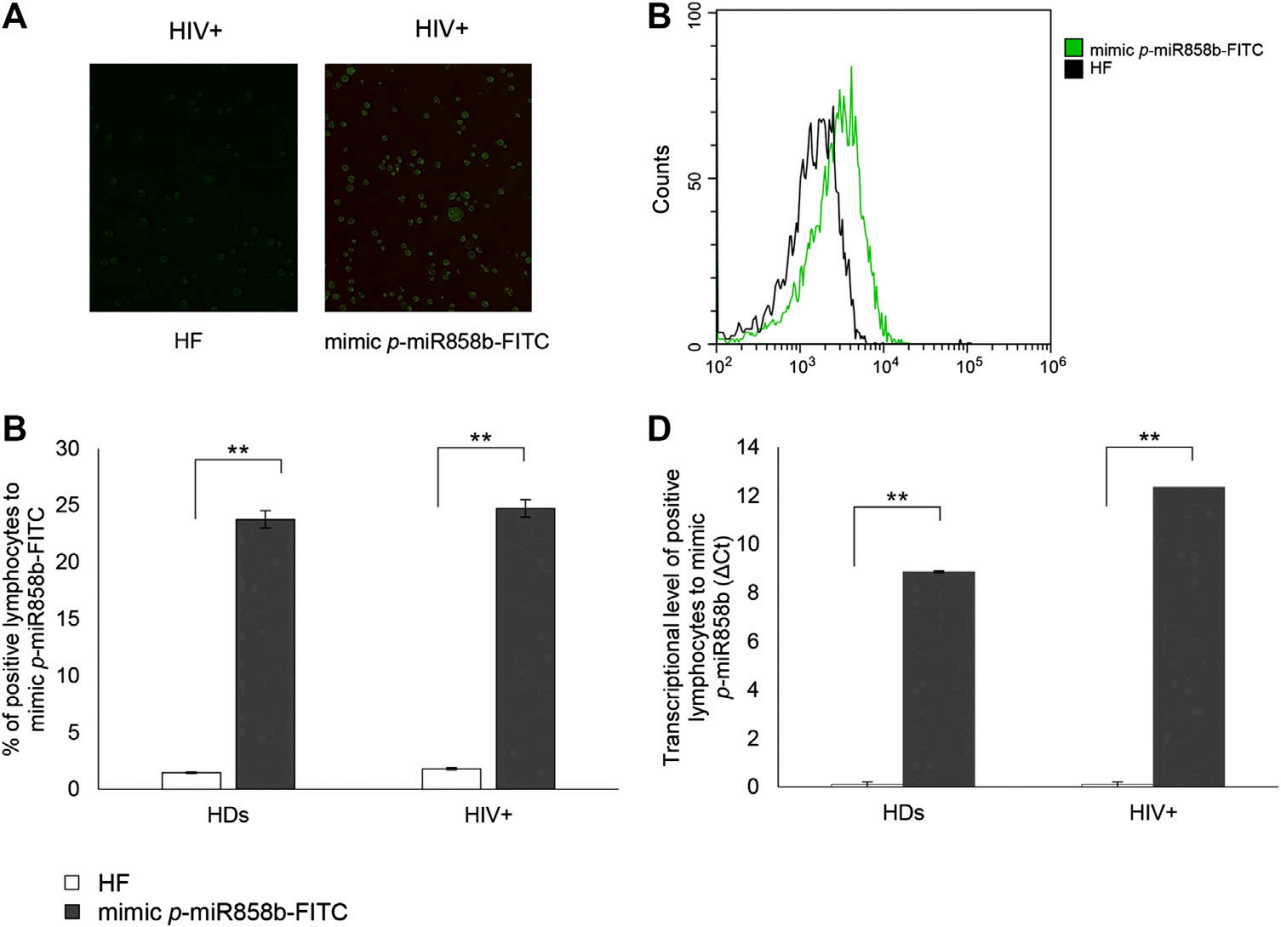
Transfection efficacy with mimic *p-*miR858b-FITC. **(A)** Representative fluorescent microscopy images of *p-*miR858b-FITC transfected lymphocytes (right panel) and its control (left panel), in 15 HIV + subjects. **(B)** Histogram overlay of transfected cells with mimic *p-*miR858b-FITC, analyzed in flow cytometry. **(C)** Percentage of HIV + lymphocytes transfected with mimic *p-*miR858-FITC. **(D)** mimic *p-*miR858b Delta Ct expression in HDs and HIV + subjects’ lymphocytes, transfected with mimic *p-*miR858b-FITC. All results derived from 15 HDs and 15 HIV + subjects **p < 0.01. All experiments were performed at least three times.

**FIGURE 8:**
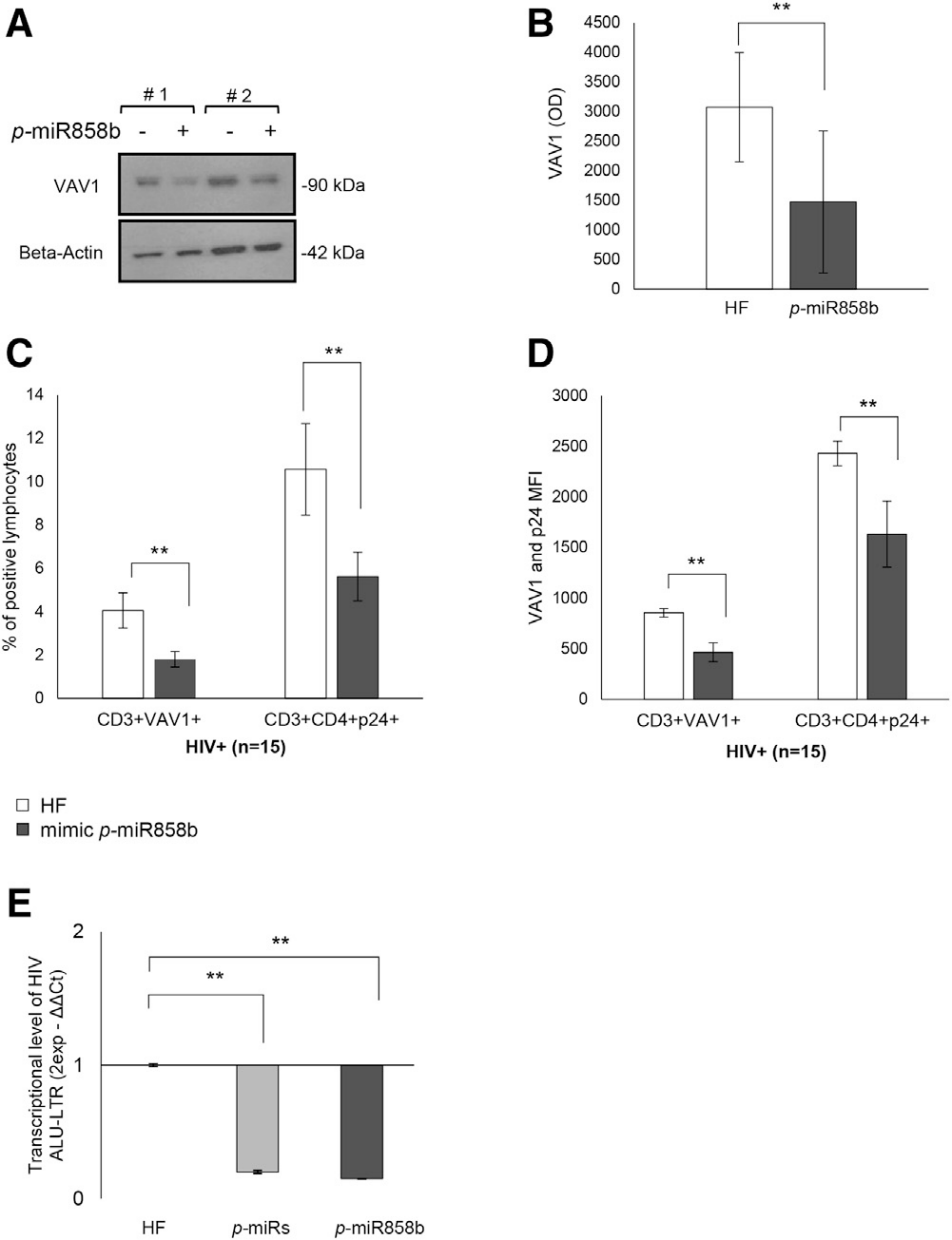
Effect of mimic *p-*miR858b transfection on VAV1 expression and HIV infection. **(A)** Representative Western Blot of two HIV+ patient of VAV1 expression level with (+) and without (-) mimic *p-*miR858b. **(B)** Densitometry histogram of the mean ± S.D. of 15 HIV+ subjects, with and without mimic *p-*miR858b. **(C)** Flow cytometry analysis of the percentage of FITC-VAV1 positive cells. **(D)** Percentage of p24^+^ cells, with and without mimic *p-*miR858b treatment. **(E)** Fold change of virus integration level, after transfection with *p*-miRs and mimic *p-*miR858b. **p < 0.01. All results derived from at least 15 HIV+ samples. The ANOVA-Bonferroni test was used.

The bioinformatics analysis highlighted that five of the analyzed *p*-miRs (*p-*miR160h, *p-*miR166, *p-*miR482b, *p-*miR395d, *p-*miR393a, and *p-*miR858b) are predicted to target VAV1. To confirm the *p*-miR-mRNA interaction, we performed the transfection on PBMCs from HIV+ naïve subjects, with a specific *p*-miR - the *p-*miR858b - verifying its ability to regulate the expression of VAV1 gene (Table 3). The *p-*miR858b was chosen for two reasons: First, among all the other *p-*miRs, it was the one which targeted the fewest genes. Second, it was part of our expertize. The human protein VAV1 is involved in HIV pathogenesis mediated by HIV Nef protein (Rauch et al., 2008), that in the early phase of the viral infection ensures T cell activation and allows the establishment of a persistent state of infection. Nef targets VAV1 and promotes its tyrosine phosphorylation, associated with its nucleus-to-cytoplasm redistribution, determining T lymphocytes activation, thus fostering virus dissemination. Transfection of mimic *p*-miR858b was performed in 15 R HIV+ patients. The transfection efficiency, analyzed by flow cytometry using a fluorescent mimic, was about 30% (Figures 7A–C), with a significant accumulation of *p*-miR858b detected by RT-PCR (Figure 7D). No specific effect on the proliferation and cell death was observed. Transfection of the PBMCs from HIV+ subjects with mimic *p*-miR858b confirmed the bioinformatics analysis, by revealing a significant decrease in the percentage of CD3^+^VAV1^+^ cells and VAV1 protein expression level (MFI). Moreover, the western blot analysis confirmed that mimic *p*-miR858b significantly inhibits the expression of VAV1 protein (Figures 8A,B). In these transfected cells, there was a significant decrease in both virus replication as demonstrated by the percentage of CD3^+^CD4^+^p24^+^ cells (Figures 8C,D), and in virus integration analysis (Figure 8E). This suggests a potential role for *p-*miR858b in the regulation of infectious mechanism associated with the expression of VAV1 in host cells. Through the RLM-RACE (RNA ligase mediated - Rapid amplification of cDNA ends) analysis, no trace of the VAV1 gene transcript was found, suggesting the regulatory action of *p-*miR858b might be at the post-transcriptional level (Supplementary Figure S2).

## Discussion

There is growing interest in the role of plant-derived miRNA in the control of several diseases, including viral infections such as HIV. This required a deeper understanding of gene networks contributing to the etiology of complex diseases. In this regard, the generated list of total genes, modulated by a pool of MO *p*-miRs to perform a functional annotation clustering, enabled us to depict the potential importance of *p*-miRs treatment by modulating a span of gene sets dysregulated in several other diseases. The wide range of disorders evidenced by the disease enrichment analysis highlighted HIV/AIDS among diseases with major burden, thereby underscoring the relevance in assessing such regulatory pathways during HIV infection. There is an urgent need to better understand such a regulatory mechanism, so unlocking its potential for enhancing HIV infection management through the use of plant-derived miRNAs. Of note, miRNAs are an important regulator of cell functions, allowing a fine-tuning at a post-transcriptional level (Bartel, 2004). These short sequences of nucleic acids are conserved across the species, and foreign miRNAs have modulatory activity in host cells, as demonstrated by interactions between plant-derived miRNAs and animal mRNAs (Zhou et al., 2015; Chin et al., 2016; Hou et al., 2018; Teng et al., 2018; Potestà et al., 2020). It is well established that plant-derived miRNAs are normally taken through the diet and are able to cross the gastrointestinal barrier, exerting a biological effect in the host. In a recent work we demonstrated that these miRNAs are vehiculated through microvesicles, allowing their delivery in the host cells (Potestà et al., 2020). These characteristics make them perfect candidates for possible therapeutic developments, compared to other small RNAs already used for the treatment of various diseases. We, therefore, focus on this Cross-Kingdom interaction to identify plant miRNA that can modulate different pathways involved in HIV pathogenesis, using PBMCs from HIV+ subjects and treated with the enriched extract of *p-*miRs from MO seeds to verify its properties. Worth noting, this cohort of HIV-individuals was chosen prior to cART initiation and within viremic individuals, in order to mitigate bias related to ART when assessing viral replication and integration mediated by *p*-miRs and specific mimics. Thus, the effects observed in the study are derived from interventions following treatment with the *p*-miRs. The putative target genes have been identified for all the most abundant *p*-miRs from the *p-*sR pool through bioinformatics analysis. This analysis supports the validation that has been highlighted in the *ex vivo* experiments, which also suggest the targets may be different, such as VAV1, TNF, BCL2, and IL2RA. Therefore, there would be diverse pathways in which these genes are involved. This underscoring the multiple regulatory capacities of the MO *p-*miR pool, since immunodeficiency in HIV cART-naïve individuals is largely due to apoptosis of CD4 T cells and to the chronic activation of the immune system. For this reason, the goals of antiviral treatment are not only to reduce viral load, but also to limit chronic activation of the immune response and lymphocytes apoptosis (Cummins and Badley, 2010). However, the reduction of apoptosis may also come at the cost of preserving the latent viral reservoirs and its increase in HIV-infected cells is desirable in this context. Our results show that the MO *p*-miRs have the ability to induce apoptosis (on both Fas- and Bcl2-mediated apoptosis) and to reduce both lymphocytes viability and viral replication. In particular, the treatment with the *p*-miR pool induces an increase of the apoptosis in lymphocytes, as demonstrated by an increment of the hypodiploid nuclei, and evidenced by an increment in Fas expression and a decrement of Bcl2 expression, compared to their control as we demonstrated earlier (Potestà et al., 2020). However, a wide distribution of apoptosis level has been observed among the HIV + individuals: the MO *p*-miR pool induced its effects, in term of apoptosis and Fas and Bcl2 expression, only in HIV + subjects with a CD4 T cell count above 200/mmc. Conversely, in HIV + subjects with a CD4 T cell count below 200/mmc no significant effect was evidenced, as well as in PBMCs from HDs. Therefore, it becomes essential to further investigate the immune-modulatory effects of *p-*miRs treatment in the two major populations of HIV+ cART-naïve individuals (Responder or Non-Responder) in real-life (Supplementary Table S2). The flow cytometry analysis of differentiation and activation markers of T lymphocytes, routinely used both in diagnosis and in the monitoring of HIV infection, highlights how the treatment modifies the differentiative pattern of CD4 T lymphocytes. Of note, declining percentage of CD4 T cell with CM (CD45RA^−^CCR7^+^) and EM (CD45RA^−^CCR7^-^) phenotype toward the TEMRA (CD45RA^+^CCR7^-^) phenotype were observed together with a decrease of activated CD25^+^ CD4^+^ lymphocytes. Similar to apoptosis, the modulation of the CD4 T cell sub-population is effective only in Responder HIV+ individuals, resulting from the long-lived resting memory CD4^+^ T cells (CM) serving as major reservoir of latent HIV infection (Zhang et al., 2019). In this context, apoptosis induced by the MO *p*-miRs in Responder HIV+ individuals may represent a powerful system to selectively induce elimination of HIV-infected CM CD4 T cell, thereby avoiding lymphocyte activation. As TNF is among the putative target of MO *p-*miRs, we also postulated that various cellular processes, such as inflammation, immune regulation or apoptosis, are mediated through binding of TNF to its receptors (Zelová and Hošek, 2013; Aquilano et al., 2019), all these processes generally being involved in HIV pathogenesis (Kumar et al., 2015; Pasquereau et al., 2017) and characterized by a high level of TNF-alpha expression (Keating et al., 2012; Paiardini and Müller-Trutwin, 2013; Planès et al., 2018). Putting this into context, our *ex vivo* system reveals that exposure to the MO *p*-miRs reduces TNF-alpha expression, thus supporting the ability of the *p-*miRs to downregulate the immune system activation also through this pathway. Analysis of the Alu sequence also shows that the treatment with both the mimic *p-*miR858b and with the MO *p*-miR pool substantially reduces viral integration. This is consistent with decline in HIV infected cells, in the frequency of p24-positive CD4 T lymphocytes, as well as reduction of their intracellular p24 protein level. Regarding the use of a specific plant miRNA, *p*-miR858b has VAV1 gene among its putative targets, so highlighting this gene’s role in T cell antigen receptor (TCR) signaling for the activation of different pathways (Tybulewicz, 2005; Ksionda et al., 2012), and for the modulation of different targets in the host cells (Abraham and Fackler, 2012; del Río-Iñiguez et al., 2018). In a context where PBMCs from HIV+ ART-naïve subjects are transfected with the mimic *p*-miR858b, the declining trend of VAV1 expression infers a decline of both viral replication and HIV DNA integration in the host cells. This regulatory ability shown by the *p-*miR858b might be due to the interaction of this miRNA with *VAV1* mRNA (Supplementary Figure S2). Interestingly, MO *p*-miR pool can reduce HIV-infected cells, albeit the mechanism behind this ability is still unclear. We therefore postulate that treatment-induced apoptosis mainly affects HIV-infected cells, reducing their number, a hypothesis reinforced by the *p-*miRs-induced decrease of CM T cells (main reservoir of latent HIV). The *p-*miRs described here exhibit a biological activity similar to that of synthetic *p-*miR858b. This evidence underscores the concept of Cross-Kingdom with specific human mRNAs and the restoration of expression in immune response and HIV control. This putative mechanism could be considered an active component in regulating the set of genes involved in the immune response against HIV. Current antiretroviral therapy recommended for HIV-infected subjects requires daily and indefinitely medication that is stressful and not without side effects, especially for younger patients. Moreover, the life-long need for therapy adherence implies high costs of chronic treatment and encourage the exploration of alternative approaches such as immunotherapy that could potentially be complementary to long-term management of HIV infection (Palma et al., 2013; Ward et al., 2020). To conclude, plant small RNAs from *Moringa oleifera* may restore normalcy in immune system and reduce replication of HIV-infection, also at the level of cellular reservoir. This suggests a role for MO *p-*miRs in standard HIV treatment, so contributing to the long-term control of the disease.

## Data Availability

The original contributions presented in the study are included in the article/Supplementary Material, further inquiries can be directed to the corresponding authors.
