# Supplementary material for: Plant microRNAs from Moringa oleifera Regulate Immune Response and HIV Infection
Source: Front Pharmacol. 2021 Feb 11;11:620038. doi: 10.3389/fphar.2020.620038 (PMC7905167; doi:10.3389/fphar.2020.620038)
Supplement: Supplementary file 1 [file datasheet1.docx]

Supplementary Material

# Supplementary Data

We selected nine different RNA-RNA interaction prediction tools, namely Miranda (Enright et al., 2003), IntaRNA 2.0 (Mann et al., 2017), RIblast (Fukunaga and Hamada, 2017), RIsearch (Wenzel et al., 2012), Probmir (Oğul et al., 2011), miRmap (Vejnar and Zdobnov, 2012), RNAplex (Tafer and Hofacker, 2008), TarpMir (Ding et al., 2016) and RNAhybrid (Rehmsmeier et al., 2004). We developed a Python code to serially run the programs and obtain as output an energy-based score (ES) and a probability-based score (PS). For each miRNA-mRNA prediction tested through these algorithms, the interaction energies and the probability values were separately combined using two scoring functions adapted from ComiR (Coronnello and Benos, 2013). In particular, the final ES score for the energies calculated by Miranda, IntaRNA, RNAplex, Tarpmir, RIsearch, RIblast and RNAhybrid has been obtained through the reformulation of the Fermi-Dirac equation (1):

| $ES =\sum_{i}^{N} \frac{1}{1+e^{{(E}_{i})/RT}}$ | (1) |
| --- | --- |

where E_i_ represents the interaction energy of the miRNA-mRNA pair and RT is the universal constant of gas. The probability scores obtained through RNAhybrid, miRmap, Tarpmir and Probmir are simply summed using the equation 2. In the case of Probmir, returning a binary classification (TARGET, NON-TARGET), a score of 1 was assigned for a positive result and 0 for a negative one.

| $PS =\sum_{i}^{N} {SCORE}_{i}$ | (2) |
| --- | --- |

The computational tool used in this work is a support vector machine (SVM) binary classifier, trained using an experimentally validated set of miRNA-mRNA interactions. Starting from the DIANA TARBASE v7 database (Vlachos et al., 2015), all interactions validated through a luciferase reporter assay experiment have been selected, obtaining 1200 positive and 380 negative interactions, respectively. The negative interactions training set was built using 200 precomputed simulated negative interactions (mock miRNAs) from the set used to train the DeepMirTar program (Wen et al., 2018). A mock miRNA is obtained by mixing its real sequence until the regions in positions from two to seven or from three to eight are no longer traceable back to the original sequence, mimicking the absence of interaction with its target. 200 interactions were extracted from the 1200 positive interactions set to build the positive training dataset. Different random set tests were generated from the remaining 1000 positive and 380 negative interactions to validate the performance of the trained SVM model. Each interaction was analysed with the prediction algorithms and the output of the scoring functions was imported in a Python environment, used as input for the *sklearn.svm.SVC* function of the *scikit-learn* package (Pedregosa et al., 2011). We selected a 2nd-degree polynomial kernel-based C-support vector classification model, enabling the option to return the vector of probabilities of belonging to each class of the response variable. The resulting classifier efficiency was evaluated using confusion matrices, precision, recall and F1 measures as implemented in the *scikit-learn metrics* library. The SVC classifier, applied to an independent test set composed by 200 positive and 200 negative interactions, correctly assigns 191 positive and 190 negative interactions, with only 10 negative and 9 positive interactions allocated to the wrong class. Analysis of precision recall and F1 measure indicates a high accuracy of the classification model, with a F1 score of 0.96 for the prediction of both negative and positive interactions. Due to the high efficiency in correctly classifying the miRNA-mRNA interactions, the classifier has been used to predict the interactions between several genes involved in cell cycle, immune response, inflammation and NfKb pathways and the *p-*miRs.


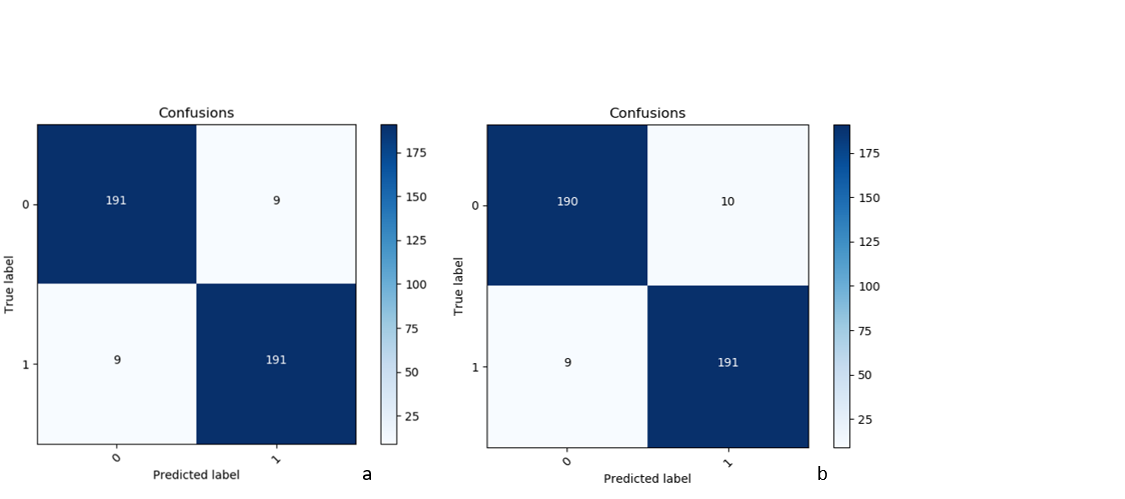


|  | **Precision** | **Recall** | **F1-score** |
| --- | --- | --- | --- |
| **Non-Target** | 0.97 | 0.95 | 0.96 |
| **Target** | 0.95 | 0.97 | 0.96 |

**SVC classifier implementation.** Result of the validation procedure carried out on and independent test set composed by 200 positive and 200 negative validated miRNA-mRNA interactions. The picture displays the obtained confusion matrix on which precision, recall, and F1 measures have been calculated.

## Supplementary Figures

**Supplementary Figure 1.** Viral load, CD4 T cells and spontaneous apoptosis. (A) Scatter plot of the Viral load (copies/ml) and CD3^+^CD4^+^ lymphocytes (cells/mmc). (B) Scatter plot of the percentage of spontaneous apoptosis and viral load (copies/ml), in HIV+ enrolled individuals (n=35 HIV+).

**Supplementary Figure 2.** (A) Relative expression level of VAV1 in PBMCs transfected with mimic *p-*miR858b, both in HDs and HIV subjects. (B) RLM-RACE VAV1 degradome for target validation. *p*-miRNA858b - conserved in many plants - was also chosen because it is present in the apple (*Malus domestica*), a plant already studied by our group for its role in the modulation of genes involved in the synthesis and accumulation of flavonoids in the fruit skin of red-fleshed apples, and for its interesting effects on human health. Moreover, bioinformatic analysis of miRNA-mRNA duplex formation reveals a high stability (Supplementary Table 3).

## Supplementary Tables

Supplementary Table 1 List of the most conserved miRNAs found in the *p-*sR pool

| *p*-miRs |
| --- |
| *p*-miR156e |
| *p*-miR159a.1 |
| *p*-miR398a-5p |
| *p*-miR397a |
| *p*-miR171b |
| *p*-miR482b |
| *p*-miR166 |
| *p*-miR396a |
| *p*-miR160h |
| *p*-miR393a |
| *p*-miR159c |
| *p*-miR2118a |
| *p*-miR858b |
| *p*-miR167f-3p |
| *p*-miR395d |

**Supplementary Table 2.** Target Genes of the *p-*miRs identified through bioinformatic analysis

|  | ***Plant-miRNA*** | ***n. targets*** | ***Genes target*** | | | |
| --- | --- | --- | --- | --- | --- | --- |
| 1 | ***miR160h*** | 68 | *ADA* (0.96) | *CD4* (0.96) | *ICOSLG* (0.96) | *IRF1* (0.98) |
|  |  |  | *AICDA* (0.95) | *CD5* (0.98) | *ICOS* (0.97) | *IRF4* (0.98) |
|  |  |  | *APC* (0.95) | *CD7* (0.96) | *IFNG* (0.95) | *LAG3* (0.96) |
|  |  |  | *BCL6* (0.98) | *CD81* (0.97) | *IL11* (0.96) | *MICB* (0.87) |
|  |  |  | *BLM* (0.98) | *CD86* (0.96) | *IL12RB1* (0.97) | *NOS2* (0.96) |
|  |  |  | *BLNK* (0.76) | *CD8A* (0.94) | *IL12RB2* (0.95) | *PTGDR2* (0.95) |
|  |  |  | *CCR1* (0.96) | *CD8B* (0.98) | *IL13* (0.95) | *SLC11A1* (0.97) |
|  |  |  | *CCR3* (0.95) | *CEBPB* (0.97) | *IL18* (0.89) | *SOCS1* (0.96) |
|  |  |  | *CCR4* (0.99) | *CSF2* (0.96) | *IL1B* (0.96) | *STAT1* (0.84) |
|  |  |  | *CCR5* (0.96) | *CX3CL1* (0.97) | *IL1R1* (0.98) | *STAT6* (0.96) |
|  |  |  | *CD274* (0.96) | *CXCR3* (0.96) | *IL1RL1* (0.98) | *TBX21* (0.97) |
|  |  |  | *CD276* (0.97) | *CXCR5* (0.97) | *IL25* (0.96) | *TGFB1* (0.96) |
|  |  |  | *CD27* (0.95) | *DPP4* (0.98) | *IL27RA* (0.95) | *TLR6* (0.96) |
|  |  |  | *CD28* (0.99) | *EBI3* (0.96) | *IL27* (0.97) | T*NFSF14* (0.92) |
|  |  |  | *CD3G* (0.96) | *EGR1* (0.92) | *IL3* (0.95) | *TNFSF4* (0.95) |
|  |  |  | *CD40LG* (0.96) | *FOXP3* (0.98) | *IL4R* (0.96) | *TNF* (0.95) |
|  |  |  | *CD40* (0.83) | *HAVCR2* (0.95) | *IL5* (0.98) | *VAV1* (0.96) |
| 2 | ***miR166*** | 50 | *AICDA* (0.96) | *CD7* (0.96) | *IL12A* (0.96) | *MAF* (0.96) |
|  |  |  | *BCL2* (0.93) | *CD81* (0.94) | *IL12RB1* (0.90) | *NFATC2* (0.96) |
|  |  |  | *BCL6* (0.95) | *CD8B* (0.98) | *IL12RB2* (0.95) | *NOS2* (0.99) |
|  |  |  | *BLNK* (0.95) | *CSF2* (0.96) | *IL13* (0.94) | *PTGDR2* (0.95) |
|  |  |  | *CCL11* (0.96) | *CX3CL1* (0.96) | *IL1R1* (0.96) | *SLC11A1* (0.96) |
|  |  |  | *CCR1* (0.95) | *CXCR3* (0.99) | *IL25* (0.97) | *SOCS1* (0.75) |
|  |  |  | *CD274* (0.98) | *CXCR5* (0.98) | *IL27RA* (0.99) | *STAT6* (0.88) |
|  |  |  | *CD276* (0.96) | *EBI3* (0.93) | *IL27* (0.98) | *TBX21* (0.98) |
|  |  |  | *CD27* (0.91) | *EGR1* (0.98) | *IL2RA* (0.87) | *TLR9* (0.95) |
|  |  |  | *CD3G* (0.95) | *ICOSLG* (0.96) | *IL4R* (0.96) | *VAV1* (0.96) |
|  |  |  | *CD40* (0.92) | *ICOS* (0.95) | *IRF1* (0.96) | *VEGFA* (0.95) |
|  |  |  | *CD4* (0.95) | *IL10* (0.99) | *IRF4* (0.79) |  |
|  |  |  | *CD5* (0.95) | *IL11* (0.95) | *LCK* (0.96) |  |
| 3 | ***miR482b*** | 38 | *AICDA* (0.97) | *CD86* (0.91) | *IL11* (0.96) | *NOS2* (0.98) |
|  |  |  | *BCL2* (0.97) | *CD8A* (0.96) | *IL12A* (0.96) | *PTGDR2* (0.96) |
|  |  |  | *CCL11* (0.95) | *CD8B* (0.98) | *IL13RA1* (0.98) | *SOCS1* (0.94) |
|  |  |  | *CCR5* (0.96) | *CX3CL1* (0.97) | *IL13* (0.96) | *STAT6* (0.96) |
|  |  |  | *CD1D* (0.89) | *CXCR5* (0.96) | *IL18R1* (0.98) | *TBX21* (0.98) |
|  |  |  | *CD276* (0.97) | *EBI3* (0.95) | *IL25* (0.97) | *TGFB1* (0.96) |
|  |  |  | *CD27* (0.95) | *EGR1* (0.98) | *IL27RA* (0.97) | *TNFSF14* (0.97) |
|  |  |  | *CD28* (0.96) | *FOXP3* (0.98) | *JAK1* (0.95) | *VAV1* (0.93) |
|  |  |  | *CD40* (0.96) | *ICOS* (0.96) | *LAG3* (0.98) |  |
|  |  |  | *CD5* (0.97) | *IL10* (0.98) | *NFATC2* (0.98) |  |
| 4 | ***miR159c*** | 26 | *CCL11* (0.96) | *EBI3* (0.96) | *IL27RA* (0.96) | *TBX21* (0.97) |
|  |  |  | *CCR5* (0.97) | *EGR1* (0.96) | *IL27* (0.80) | *TGFB1* (0.98) |
|  |  |  | *CD2* (0.95) | *GFI1* (0.93) | *IL2RA* (0.98) | *TLR6* (0.97) |
|  |  |  | *CD40LG* (0.98) | *HAVCR2* (0.97) | *IL4R* (0.84) | *TNF* (0.98) |
|  |  |  | *CD8A* (0.91) | *ICOS* (0.79) | *NOS2* (0.89) | *VEGFA (0.92)* |
|  |  |  | *CX3CL1* (0.95) | *IL10* (0.97) | *SLC11A1* (0.97) |  |
|  |  |  | *CXCR5* (0.86) | *IL1B* (0.98) | *SOCS1* (0.95) |  |
| 5 | ***miR395d*** | 23 | *ADA* (0.96) | *CD81* (0.96) | *IRF1* (0.92) | *TBX21* (0.95) |
|  |  |  | *BCL2* (0.91) | *CXCR5* (0.87) | *MICB* (0.77) | *TGFB1* (0.98) |
|  |  |  | *BLNK* (0.96) | *HAVCR2* (0.94) | *NOS2* (0.98) | *TLR1* (0.98) |
|  |  |  | *CD3G* (0.98) | *IL10* (0.97) | *PTGDR2* (0.98) | *VAV1* (0.96) |
|  |  |  | *CD4* (0.97) | *IL27* (0.97) | *RIPK2* (0.96) |  |
|  |  |  | *CD7* (0.87) | *IL3* (0.96) | *STAT1* (0.95) |  |
| 6 | ***miR159a.1*** | 20 | *CCL11* (0.98) | *CD2* (0.95) | *GATA3* (0.98) | *IL27* (0.88) |
|  |  |  | *CCR5* (0.98) | *CD40LG* (0.98) | *HAVCR2* (0.98) | *IL4R* (0.98) |
|  |  |  | *CD1D* (0.96) | *CX3CL1* (0.95) | *IL10* (0.98) | *NFATC2* (0.83) |
|  |  |  | *CD274* (0.71) | *EBI3* (0.96) | *IL12A* (0.95) | *SLC11A1* (0.97) |
|  |  |  | *CD28* (0.98) | *EGR1* (0.96) | *IL27RA* (0.96) | *SOCS1* (0.95) |
| 7 | ***miR2118a*** | 15 | *CCL11* (0.95) | *DPP4* (0.98) | *IL13RA1* (0.89) | *TLR6* (0.95) |
|  |  |  | *CD40* (0.73) | *FOXP3* (0.95) | *IL1RL1* (0.94) | *TNFSF14* (0.95) |
|  |  |  | *CXCR3* (0.79) | *ICOSLG* (0.96) | *IL27* (0.95) | *TNF* (0.90) |
|  |  |  | *CXCR5* (0.83) | *IL11* (0.94) | *IRF1* (0.95) |  |
| 8 | ***miR393a*** | 11 | *BLNK* (0.96) | *EGR1* (0.96) | *JAK1* (0.99) | *SOCS1* (0.98) |
|  |  |  | *CD3E* (0.97) | *IL25* (0.97) | *PTGDR2* (0.95) | *VAV1* (0.88) |
|  |  |  | *CD5* (0.83) | *IL3* (0.84) | *SLC11A1* (0.94) |  |
| 9 | ***miR167f-3p*** | 10 | *CD2* (0.96) | *CXCR3* (0.81) | *IRF1* (0.98) | *TNF* (0.94) |
|  |  |  | *CD40LG* (0.98) | *FASLG* (0.94) | *PTGDR2* (0.96) | *STAT6* (0.81) |
|  |  |  | *CSF2* (0.95) | *IL11* (0.95) | *TLR9* (0.95) |  |
| 10 | ***miR396a*** | 8 | *CCL11* (0.99) | *CD81* (0.95) | *IL10* (0.72) | *IL1RL1* (0.96) |
|  |  |  | *CCR2* (0.95) | *DPP4* (0.96) | *IL11* (0.81) | *JAK1* (0.96) |
| 11 | ***miR858b*** | 6 | *BLNK* (0.96) | *IL18* (0.82) | *TBX21* (0.98) |  |
|  |  |  | *IL11* (0.84) | *IRF1* (0.98) | *VAV1* (0.95) |  |
| 12 | ***miR156e*** | 4 | *CD81* (0.97) | *FOXP3* (0.98) | *IL1R1* (0.99) | *TNFSF4* (0.97) |
| 13 | ***miR398a-5p*** | 4 | *CCR2* (0.96) | *CCR3* (0.97) | *IL3* (0.95) | *TBX21* (0.92) |
| 14 | ***miR171b*** | 4 | *ADA* (0.96) | *CSF2* (0.86) | *IL12RB1* (0.90) | *IL15* (0.86) |
| 15 | ***miR397a*** | 3 | *CD7* (0.90) | *FASLG* (0.97) | *TLR9* (0.91) |  |

| **Supplementary Table 3.** Bioinformatic analysis by MIRANDA (miRNA-mRNA duplex formation) | | | | | |
| --- | --- | --- | --- | --- | --- |
|  | ***Alignment 3’ → 5’*** | ***Homology (%)*** | ***mRNA-miRNA*** | | ***Free Energy variation (DG)*** |
| 3’UTR | ag TCCAGCT T G- TCTG TTGCT t  cg AGGTCAAGCGAGACAACGAg | 70% | human *VAV1* mRNA  *mdo*-miR858b | -27,07 (kcal/mol) | |

## Supplementary References

Coronnello, C., and Benos, P. V. (2013). ComiR: combinatorial microRNA target prediction tool. *Nucleic Acids Res.* 41, W159–W164. doi:10.1093/nar/gkt379.

Ding, J., Li, X., and Hu, H. (2016). TarPmiR: a new approach for microRNA target site prediction. *Bioinformatics* 32, 2768–75. doi:10.1093/bioinformatics/btw318.

Enright, A. J., John, B., Gaul, U., Tuschl, T., Sander, C., and Marks, D. S. (2003). MicroRNA targets in Drosophila. *Genome Biol.* 5, R1. doi:10.1186/gb-2003-5-1-r1.

Fukunaga, T., and Hamada, M. (2017). RIblast: an ultrafast RNA-RNA interaction prediction system based on a seed-and-extension approach. *Bioinformatics* 33, 2666–2674. doi:10.1093/bioinformatics/btx287.

Mann, M., Wright, P. R., and Backofen, R. (2017). IntaRNA 2.0: enhanced and customizable prediction of RNA–RNA interactions. *Nucleic Acids Res.* 45, W435–W439. doi:10.1093/nar/gkx279.

Oğul, H., Umu, S. U., Tuncel, Y. Y., and Akkaya, M. S. (2011). A probabilistic approach to microRNA-target binding. *Biochem. Biophys. Res. Commun.* 413, 111–115. doi:10.1016/J.BBRC.2011.08.065.

Pedregosa, F., Varoquaux, G., Gramfort, A., Michel, V., Bertrand, T., Grisel, O., et al. (2011). Scikit-learn: Machine Learning in Python. Available at: http://scikit-learn.sourceforge.net. [Accessed April 16, 2019].

Rehmsmeier, M., Steffen, P., Hochsmann, M., and Giegerich, R. (2004). Fast and effective prediction of microRNA/target duplexes. *RNA* 10, 1507–17. doi:10.1261/rna.5248604.

Tafer, H., and Hofacker, I. L. (2008). RNAplex: a fast tool for RNA–RNA interaction search. *Bioinformatics* 24, 2657–2663. doi:10.1093/bioinformatics/btn193.

Vejnar, C. E., and Zdobnov, E. M. (2012). miRmap: Comprehensive prediction of microRNA target repression strength. *Nucleic Acids Res.* 40, 11673–11683. doi:10.1093/nar/gks901.

Vlachos, I. S., Zagganas, K., Paraskevopoulou, M. D., Georgakilas, G., Karagkouni, D., Vergoulis, T., et al. (2015). DIANA-miRPath v3.0: deciphering microRNA function with experimental support. *Nucleic Acids Res.* 43, W460-6. doi:10.1093/nar/gkv403.

Wen, M., Cong, P., Zhang, Z., Lu, H., and Li, T. (2018). DeepMirTar: a deep-learning approach for predicting human miRNA targets. *Bioinformatics* 34, 3781–3787. doi:10.1093/bioinformatics/bty424.

Wenzel, A., Akbaşli, E., and Gorodkin, J. (2012). RIsearch: Fast RNA-RNA interaction search using a simplified nearest-neighbor energy model. *Bioinformatics* 28, 2738–2746. doi:10.1093/bioinformatics/bts519.
